# HIV PrEP is more than ART‐lite: Longitudinal study of real‐world PrEP services data identifies missing measures meaningful to HIV prevention programming

**DOI:** 10.1002/jia2.25827

**Published:** 2021-10-14

**Authors:** Jason Bailey Reed, Prakriti Shrestha, Daniel Were, Tafadzwa Chakare, Jane Mutegi, Brian Wakhutu, Abednego Musau, Nyane Matebello Nonyana, Alice Christensen, Rupa Patel, Jessica Rodrigues, Robyn Eakle, Kelly Curran, Diwakar Mohan

**Affiliations:** ^1^ Jhpiego Baltimore Maryland USA; ^2^ Department of International Health Johns Hopkins Bloomberg School of Public Health Baltimore Maryland USA; ^3^ Jhpiego Nairobi Nairobi Kenya; ^4^ Jhpiego Maseru Maseru Lesotho; ^5^ Jhpiego Dar es Salaam Tanzania; ^6^ Washington University St. Louis Missouri USA; ^7^ AVAC New York City New York USA; ^8^ USAID Washington DC USA

**Keywords:** adherence, continuation, HIV pre‐exposure prophylaxis, retention, sub‐Saharan Africa

## Abstract

**Introduction:**

Evidence indicates HIV oral pre‐exposure prophylaxis (PrEP) is highly efficacious and effective. Substantial early discontinuation rates are reported by many programs, which may be misconstrued as program failure. However, PrEP use may be non‐continuous and still effective, since HIV risk fluctuates. Real‐world PrEP use phenomena, like restarting and cyclical use, and the temporal characteristics of these use patterns are not well described. The objective of our study was to characterize and identify predictors of use patterns observed in large PrEP scale‐up programs in Africa.

**Methods:**

We analysed demographic and clinical data routinely collected during client visits between 2017 and 2019 in three Jhpiego‐supported programs in Kenya, Lesotho and Tanzania. We characterized duration on/off PrEP and, using ordinal regression, modelled the likelihood of spending additional time off and identified factors associated with increasing cycle number. The Andersen‐Gill model was used to identify predictors of time to PrEP discontinuation. To analyse factors associated with a client's first return following initiation, we used a two‐step Heckman probit.

**Results:**

Among 47,532 clients initiating PrEP, approximately half returned for follow‐up. With each increase in cycle number, time off PrEP between use cycles decreased. The Heckman first‐step model showed an increased probability of returning versus not by older age groups and among key and vulnerable population groups versus the general population; in the second‐step model older age groups and key and vulnerable populations were less likely in Kenya, but more likely in Lesotho, to return on‐time (refill) versus delayed (restarting).

**Conclusions:**

PrEP users frequently cycle on and off PrEP. Early discontinuation and delays in obtaining additional prescriptions were common, with broad predictive variability noted. Time off PrEP decreased with cycle number in all countries, suggesting normalization of use with experience. More nuanced measures of use are needed than exist for HIV treatment if effective use of PrEP is to be meaningfully measured. Providers should be equipped with measures and counselling messages that recognize non‐continuous and cyclical use patterns so that clients are supported to align fluctuating risk and use, and can readily restart PrEP after stopping, in effect empowering them further to make their own prevention choices.

## 1 INTRODUCTION

HIV oral pre‐exposure prophylaxis (PrEP) was shown in clinical trials to be highly efficacious in preventing HIV infection among those with higher drug concentrations in blood and tissues, indicative of proximal PrEP adherence [1, 2]. The World Health Organization (WHO) recommends no particular use duration, instead noting that use should cover “seasons” of substantial risk [3]. In an effort to optimize PrEP's epidemic impact and individual wellbeing, national programs have stressed clients’ daily adherence to the dosing regimen prescribed and persistent use over time.

Many programs prescribing daily oral PrEP have noted high rates of discontinuation within the first month of use, based upon observed delays in prescription refills [4]. Published modelling studies suggest diminishing impact and cost effectiveness as use duration decreases [5, 6], leading to speculations about whether clients’ ability and willingness to use PrEP “long enough” may prove too difficult to make it a feasible and strategic HIV prevention intervention in low‐ and middle‐income country settings [7, 8]. However, PrEP users have reported risk‐use alignment, which refers to non‐continuous dosing corresponding to episodic risk that often spans days or weeks rather than consecutive, continuous months [9, 10, 11, 12, 13, 14, 15]. There is early evidence of reduced HIV incidence even in the context of clients’ early and frequent PrEP discontinuation [16, 17, 18], which points to a need for appropriate use metrics to more fully describe use patterns, especially non‐continuous dosing, to ensure PrEP use remains as effective as possible [19, 20].

Monitoring and evaluation (M&E) indicators for HIV PrEP set forth by donors and normative agencies have largely mirrored HIV antiretroviral therapy (ART) indicators, focusing on continuous, prolonged use. The WHO's PrEP adherence indicator specifies the measurement of continued use at 3 months after starting [21], suggesting a minimum effective use period, far beyond the duration of use of many clients. The US President's Emergency Plan for AIDS Relief's (PEPFAR) PrEP indicators similarly measure use spanning a minimum of calendar quarters, mirroring their HIV ART retention indicator and aligned with WHO [21]. Unlike HIV treatment, however, where any interruption in use poses a risk of adverse outcomes, for example, viral rebound, interruption in PrEP use may pose no risk [14]. Stopping (and restarting) may reflect clients’ decisions to switch prevention methods or their determination that their risk has changed, as cited in published literature [9, 10, 22]. Thus, messaging and monitoring PrEP as a time‐limited option for HIV prevention that will likely be stopped and restarted as needed, instead of as an intervention like ART that requires lifelong and continuous use, may remove reported barriers to use [19, 20, 23]. That said, clients’ initial experience using PrEP may influence subsequent use/use effectiveness, so supporting normalization of first use may be a useful investment in future effective use. Normalization of early medication use has been linked with more stable longer‐term use for oral contraceptives, post‐transplant drug regimens, and ART [24, 25, 26, 27, 28, 29], and it reasons that the same could apply to oral PrEP since it also requires a degree of repetitive use.

To better assess effective PrEP programming in the early years of execution, it is critical to better understand use phenomena, such as early stopping, delays to refilling prescriptions and gaps between stopping and restarting PrEP in the context of on‐going or resumed risk. In our study, we use such metrics to describe characteristics and predictors of continuous versus cyclical PrEP use, as well as restarts of PrEP after stopping for varying durations. These analyses are expected to help expand stakeholders’ understanding of PrEP use in real‐world settings, specifically as distinct from HIV treatment, and understand critical metrics to inform management strategies.

## 2 MATERIALS AND METHODS

This study used client‐level longitudinal data of 47,532 clients who were 15 years of age or older and who started daily oral PrEP (not event‐driven PrEP), customarily dispensed as a 30‐pill/1‐month supply, between as early as 2017 and as late as 2019 in three Jhpiego‐supported programs in Kenya, Tanzania and Lesotho. Demographic, clinical and prescription data from all three countries were routinely collected at each client visit using a clinical form or format (if electronically collected) approved by each countries’ health ministry. Data collected on paper were entered into an electronic database. Further details regarding data collection are available in a previous publication [30]. The data presented in this manuscript received a non‐human subjects research determination by the Johns Hopkins School of Public Health institutional review board (JHSPH IRB) No. 00008634. All analyses were secondary analyses of the de‐identified, routine service delivery data covered by the JHSPH IRB research determination above; thus, consent was not required.

In our analysis, we included clients with at least 3 months of observation time, which would allow them sufficient opportunity to return for at least one follow‐up PrEP prescription. In Kenya and Tanzania, clients had initiated PrEP before December 2019 and were observed through February 2020. In Lesotho, clients had initiated PrEP before June 2019 and were observed until September 2020. In Kenya, the Jilinde PrEP program reaches at‐risk populations through both public and private service models including drop‐in‐centres designed as one‐stop‐shops for the needs of specific sub‐populations. Tanzania's Sauti program was a comprehensive sexual and reproductive health program that included PrEP delivered to female sex workers (FSW) through brothel‐based services. Lesotho's Technical Support to Enhance HIV/AIDS Prevention and Opportunities in Nursing Education (TSEPO) was a program that provided PrEP primarily to adolescent girls and young women (AGYW) through community venues.

### 2.1 Variables

The demographic variables collected at the baseline visit included age, sex, marital status and risk population group, including men who have sex with men (MSM); FSW; HIV‐negative partner in a serodiscordant relationship (SDC); AGYW; apart from those clients categorized as the general population. We captured data recorded by clinicians on clients’ history of previous PrEP use and any sexually transmitted infection (STI) diagnosed at the first visit to the program.

**Figure 1:**
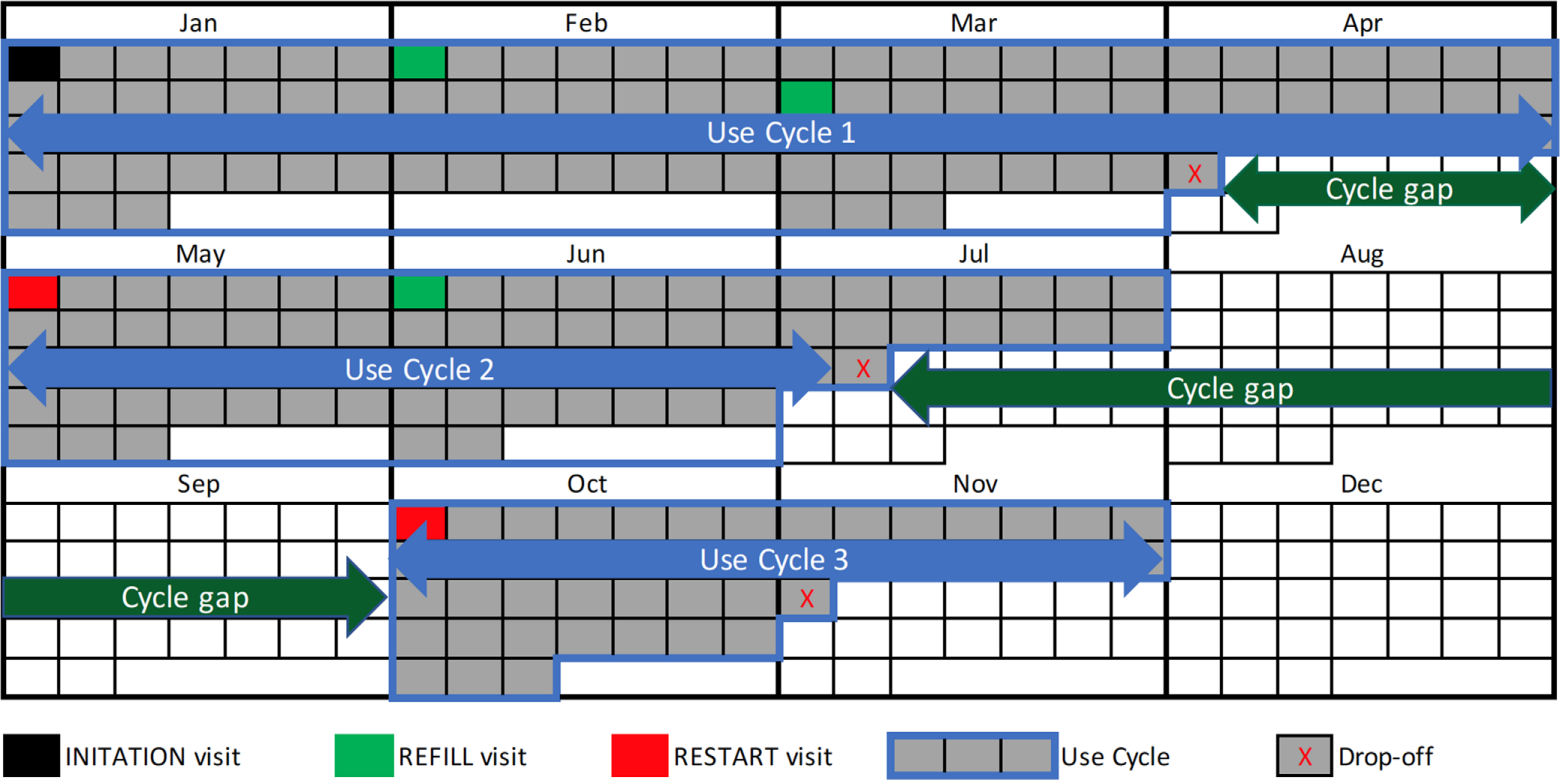
Visual calendar‐year outline of an illustrative individual client's use and non‐use durations of PrEP to depict study terminology. The use cycle is depicted by the shaded grey boxes (calendar days), which sum to total days’ duration of use for each (cycle); the span of use is represented visually by the light blue arrow. The cycle gap is depicted by the unshaded white boxes (again, calendar days), which sum to the total day's duration of non‐use between each (cycle); the span of non‐use is represented visually by the dark green arrow

A client's “initiation” visit was defined as the client's first PrEP prescription from the program. A “refill” visit referred to an on‐time return for additional PrEP supply, defined as a return within 14 days of the calculated date a client would no longer have PrEP tablets. PrEP discontinuation or “drop‐off” was defined as a delay of 15 or more days in returning for a follow‐on prescription (or no return at all). A client's return visit after drop‐off was referred to as a “restart.” The duration in days between a drop‐off and the next restart date was referred to as the “cycle gap.” The initiation prescription and subsequent restart(s) of PrEP defined the beginning of independent “use cycle(s),” with each cycle continuing for as long as refills were obtained without delay. The “time observed in program” variable was defined as the number of days between a client's first visit and the end of the observation period in the analysis. The “time observed through last cycle’ variable was defined as the number of days between a client's first visit and the date of apparent last use after the last visit in each cycle. These terms are outlined visually in Figure 1, and in Supporting information Table S1. Thus, all clients had the initiation prescription, and thereafter could have had any number of refill and/or restart prescriptions. In turn, each client could have any number of use cycles, each potentially varying in days’ duration.

We specifically categorized our analyses into two main sub‐sections. The first reviewed all cycles of use to obtain a comprehensive understanding of clients’ longitudinal use of PrEP. The second focused solely on clients’ first cycle of PrEP use with the program. The findings from the two analyses serve different purposes. The first analyses intend to inform providers about better counselling clients on stopping and restarting to ensure non‐continuous use is as effective as possible. The second analyses intend to better characterize only initial use since evidence suggests normalizing early medication use may ensure ease of future use. We present all analyses by country to account for between‐country variations.

### 2.2 Statistical analysis

#### 2.2.1 Descriptive analyses

Because of the non‐normal distribution of the days of duration of PrEP use and clients’ overall time in the program since their initiation, we used medians and interquartile ranges to summarize the data. We used frequencies to describe the clients’ sample characteristics and the patterns of use for those who returned following initiation.

#### 2.2.2 Ordinal regression

We used ordinal logistic regression to analyse factors associated with the likelihood of spending time (in months) off PrEP (cycle gap) between the first and second use, and between all cycles. In addition, we used ordinal regression to identify factors associated with the total number of use cycles per client.

#### 2.2.3 Andersen–Gill model

For our analysis of all PrEP use cycles among clients, we used the Andersen‐Gill model, an extension of Cox regression modelling, to identify clinical and demographic predictors of time to drop‐off or the time elapsed to stopping PrEP after the initial start, adjusted for the time observed through last cycle, which is the number of days a client had spent in the program at the end of each cycle [31, 32]. In our study, the event of interest (drop‐off) can occur multiple times and is described as a recurrent event. Andersen–Gill allows for the analysis of the intensity of a recurrent event and time‐dependent covariates [32]. Each client could have contributed multiple cycles with time to drop‐off to the analysis, and Andersen‐Gill modelling allows for the specification of clients on PrEP and off PrEP. We used a robust variance estimator to account for the assumptions that each event was independent of each other. Kaplan–Meier curves were used to explore the clients’ time to drop‐off from a cycle over the time in the program and are presented as Supporting information Figures S1 and 2.

#### 2.2.4 Heckman Probit model

To correct the sample selection bias resulting from clients who did not return at all after their initiation visit, we used a two‐step Heckman Probit model for the data analyses to analyse factors associated with clients returning for a refill versus a restart after their initiation visit. The first step estimates the probability of returning versus not, while the second step estimates the probability of refilling versus restarting, correcting for the self‐selection that the client returns. Heckman Probit estimates are presented with the 95% Confidence Intervals (CI). The probit estimates from the Heckman models are presented for Kenya and Lesotho, while the lack of variation in covariates prevented model convergence for Tanzania. All analyses were performed using Stata 16.0 (College Station, Texas) [33].

## 3 RESULTS

During the observation period between 2017 and 2019, 47,532 clients started taking PrEP: 32,963 in Kenya; 8510 in Lesotho and 6059 in Tanzania. Across the three countries, the majority were female (77.1%), 20–24 years of age (33.5%), FSW (49.6%), and not currently married (76.8%). Since different programs purposefully prioritized different risk population groups per donor‐specified targets, characteristics of PrEP clients varied accordingly across countries. Sample characteristics by country are presented in Table 1a, 1b, 1c. The median number of days from initiation to the end of the observation period was 628 days (IQR: 378–830) in Kenya, 575 days (IQR: 447–610) in Tanzania and 349 days (IQR: 243–440) in Lesotho. The median duration of PrEP use was 30 days (IQR: 30,60) overall, 30 days (IQR: 30–61) in Kenya, 30 days (IQR: 30–40) in Lesotho, and 30 days (IQR: 30–60) in Tanzania.

**Table 1a:** Demographic characteristics of clients in Kenya/Jilinde

		Year of first visit			
Factor	Level	All years	2017	2018	2019
**N**		32,963	9585	13,515	9863
Age, median (IQR)		25.0 (21.0–30.0)	24.0 (21.0–30.0)	25.0 (21.0–30.0)	25.0 (21.0–31.0)
Age categories	15–19	4887 (14.8%)	1477 (15.4%)	1742 (12.9%)	1668 (16.9%)
	20–24	11,183 (33.9%)	3346 (34.9%)	4606 (34.1%)	3231 (32.8%)
	25–29	7465 (22.6%)	2226 (23.2%)	3316 (24.5%)	1923 (19.5%)
	30–34	4652 (14.1%)	1264 (13.2%)	1976 (14.6%)	1412 (14.3%)
	35 and over	4776 (14.5%)	1272 (13.3%)	1875 (13.9%)	1629 (16.5%)
Sex^§^	Male	8136 (24.7%)	2584 (27.0%)	3274 (24.2%)	2278 (23.1%)
	Female	24,826 (75.3%)	7000 (73.0%)	10,241 (75.8%)	7585 (76.9%)
Marital Status	Not Married	24,035 (72.9%)	7299 (76.2%)	10,015 (74.1%)	6721 (68.1%)
	Currently Married	8928 (27.1%)	2286 (23.8%)	3500 (25.9%)	3142 (31.9%)
Risk Population Group	GEN POP	4352 (13.2%)	1559 (16.3%)	1495 (11.1%)	1298 (13.2%)
	MSM	4780 (14.5%)	1667 (17.4%)	2085 (15.4%)	1028 (10.4%)
	FSW	17,480 (14.5%)	5335 (55.7%)	7980 (59.0%)	4165 (42.2%)
	SDC	3658 (11.1%)	841 (8.8%)	1479 (10.9%)	1338 (13.6%)
	AGYW	2693 (8.2%)	183 (1.9%)	476 (3.5%)	2034 (20.6%)
Prior PrEP Use	No	31,107 (96.5%)	9007 (96.1%)	12,693 (96.1%)	9407 (97.4%)
	Yes	1135 (3.5%)	363 (3.9%)	518 (3.9%)	254 (2.6%)
STI diagnosis at Visit 1	No	32,190 (99.4%)	9338 (99.4%)	13,196 (99.2%)	9656 (99.5%)
	Yes	206 (0.6%)	52 (0.6%)	107 (0.8%)	47 (0.5%)

^§^ There was one transgender client (<1%).

IQR, interquartile range; STI, sexually transmitted infection.

**Table 1b:** Demographic characteristics of clients in Lesotho/TSEPO

		Year of first visit			
Factor	Level	All years	2017	2018	2019
**N**		8510	6	5855	2649
Age, median (IQR)		23.0 (19.0–29.0)	20.5 (19.0–31.0)	24.0 (20.0–31.0)	21.0 (18.0–24.0)
Age categories	15–19	2254 (26.5%)	3 (50.0%)	1253 (21.4%)	998 (37.7%)
	20–24	2830 (33.2%)	1 (16.7%)	1773 (30.3%)	1056 (39.9%)
	25–29	1490 (17.5%)	0 (0.0%)	1178 (20.1%)	312 (11.8%)
	30–34	880 (10.3%)	2 (33.3%)	734 (12.5%)	144 (5.4%)
	35 and over	1056 (12.4%)	0 (0.0%)	917 (15.7%)	139 (5.2%)
Sex	Male	2546 (29.9%)	1 (16.7%)	2212 (37.8%)	333 (12.6%)
	Female	5964 (70.1%)	5 (83.3%)	3643 (62.2%)	2316 (87.4%)
Marital status	Not Married	6395 (84.0%)	7 (100.0%)	4299 (85.4%)	2110 (81.3%)
	Currently Married	1217 (16.0%)	0 (0.0%)	733 (14.6%)	486 (18.7%)
Risk population group	GEN POP	3765 (44.2%)	3 (42.9%)	3077 (52.5%)	685 (25.8%)
	MSM	398 (4.7%)	0 (0.0%)	308 (5.3%)	90 (3.4%)
	FSW	364 (4.3%)	0 (0.0%)	291 (5.0%)	73 (2.7%)
	SDC	259 (3.0%)	0 (0.0%)	222 (3.8%)	37 (1.4%)
	AGYW	3724 (43.8%)	3 (57.1%)	1957 (33.5%)	1764 (66.7%)
Prior PrEP use	No	8510 (100.0%)	6 (100.0%)	5855 (100.0%)	2649 (100.0%)
	Yes	0 (0.0%)	0 (0.0%)	0 (0.0%)	0 (0.0%)
STI diagnosis at visit 1	No	5101 (60.0%)	6 (100.0%)	3597 (61.4%)	1498 (56.6%)
	Yes	3407 (40.0%)	0 (0.0%)	2258 (38.6%)	1149 (43.4%)

IQR, interquartile range; STI, sexually transmitted infection.

**Table 1c:** Demographic characteristics of clients in Tanzania/Sauti

		Year of first visit		
Factor	Level	All years	2018	2019
**N**		6059	4570	1489
Age, median (IQR)		26.0 (22.0–32.0)	26.0 (22.0–31.0)	27.0 (22.0–33.0)
Age categories	15–19	619 (10.2%)	459 (10.0%)	160 (10.7%)
	20–24	1817 (30.0%)	1414 (30.9%)	403 (27.1%)
	25–29	1620 (26.7%)	1218 (26.7%)	402 (27.0%)
	30–34	957 (15.8%)	733 (16.0%)	224 (15.0%)
	35 and over	1046 (17.3%)	746 (16.3%)	300 (20.1%)
Sex	Male	434 (7.2%)	0 (0.0%)	434 (29.1%)
	Female	5625 (92.8%)	4570 (100.0%)	1055 (70.9%)
Marital status	Not Married	5878 (99.7%)	4550 (99.6%)	1328 (100.0%)
	Currently Married	20 (0.3%)	20 (0.4%)	0 (0.0%)
Risk population group	MSM	434 (7.2%)	0 (0.0%)	434 (29.1%)
	FSW	5624 (92.8%)	4570 (100.0%)	1054 (70.8%)
	AGYW	1 (<1%)	0 (0.0%)	1 (0.1%)
Prior PrEP use	No	6059 (100.0%)	4570 (100.0%)	1489 (100.0%)
	Yes	0 (0.0%)	0 (0.0%)	0 (0.0%)
STI diagnosis at visit 1	No	159 (2.6%)	0 (0.0%)	159 (10.7%)
	Yes	5900 (97.4%)	4570 (100.0%)	1330 (89.3%)

IQR, interquartile range; STI, sexually transmitted infection.

Among 47,532 clients, 21,303 (44.8%) returned one or more times for additional PrEP supply, while 26,229 (55.2%) had not returned at all by the end of the observation period (Table 2). Of the 21,303 clients who did return, 14,143 (66.4%) received the first follow‐on prescription on time, a “refill”; this is in comparison to 7160 (33.6%) clients who were delayed for their first return and thus “restarted” on PrEP as a new use cycle. When evaluating restarts, 2394 (33.4%), 929 (13.0%) and 764 (10.7%) experienced 2, 3, and 4 or more restarts, respectively. The elapsed duration between the first PrEP stop and restart is outlined in Table 3 for all three countries as well as by each country.

**Table 2:** Follow‐up status after initiating PrEP

	Total no. of clients—N	No Return n (%)	Refill n(%)	Restart n(%)
Kenya	32,963	16,406 (49.8%)	11,069 (33.6%)	5488 (16.7%)
Lesotho	8510	6078 (71.4%)	2011 (23.6%)	421 (5.0%)
Tanzania	6059	3745 (61.8%)	1063 (17.5%)	1251 (20.7%)

**Table 3:** Number of days between first PrEP stop (drop‐off) and restart by country

Elapsed duration between PrEP stop and restart	No. of clients, n (%)	Kenya, n (%)	Lesotho, n (%)	Tanzania, n (%)
15–30 days	2486 (20.8%)	2104 (21.6%)	222 (30.9%)	160 (10.7%)
30–60 days	3286 (27.5%)	2783 (28.6%)	185 (25.8%)	318 (21.4%)
61–90 days	2040 (17.1%)	1404 (14.5%)	88 (12.3%)	548 (36.7%)
91–180 days	2080 (17.4%)	1647 (16.9%)	95 (13.2%)	340 (22.8%)
≥180 days	2043 (17.1%)	1789 (18.4%)	128 (17.8%)	126 (8.4%)

There were variations across countries in proportions refilling versus restarting PrEP, which are further described in Table 2. Notably, 16,557 (50.2%) of clients returned one or more times for additional PrEP supply in Kenya, while only 2432 (28.6%) and 2314 (38.2%) returned in Lesotho and Tanzania, respectively (Table 2). Among those who returned, the majority returned on‐time for refills in both Kenya (66.8%) and Lesotho (82.7%), while only 45.9% of clients returned for an on‐time refill in Tanzania (Table 2). The maximum observed number of refills in sequence for a client was 32 refills in Kenya, and 6 refills in both Lesotho and Tanzania. Four‐hundred and eighty‐six, 86 and zero clients had a single‐use cycle comprised of continuous (on‐time) refills throughout the entirety of their observation period, in Kenya, Lesotho and Tanzania, respectively, noting again the period of observation varied by client. The maximum observed number of restarts for a client was 10 in Kenya, 4 in Lesotho and 3 in Tanzania.

### 3.1 All cycles

Using the results of the ordinal regression model (Table 4a, 4b, 4c), independent predictors of an increased number of PrEP use cycles in Kenya included being older (**≥**20 years), and MSM, FSW, or SDC. In Lesotho, being older (**≥**30 years), MSM, FSW, or AGYW, and having a positive STI diagnosis at baseline were associated with an increased number of PrEP use cycles (Table 4a, 4b). In Tanzania, only female clients were associated with an increased number of PrEP use cycles (Table 4c).

**Table 4a:** Crude and adjusted ordinal regression of factors associated with cycle number (Kenya/Jilinde)

	No. of clients	cOR [95% CI]	aOR [95% CI]
Time observed in program	32,963	1.003^***^[1.002,1.003]	1.003^***^[1.003,1.003]
Cycle length	32,963	1.004^***^[1.003,1.004]	1.001^**^[1.000,1.001]
Age			
15—19	4887	REF	REF
20—24	11,183	1.289^***^[1.192, 1.393]	1.268^***^[1.134,1.419]
25‐=29	7464	1.709^***^[1.575,1.855]	1.727^***^[1.527,1.954]
30—35	4652	1.821^***^[1.665,1.991]	2.221^***^[1.931,2.554]
35 and over	4776	2.079^***^[1.904,2.271]	2.640^***^[2.307,3.021]
Sex			
Male	8136	REF	REF
Female	24,825	1.028 [0.974,1.086]	1.114 [0.955,1.300]
Marital status			
Not married	24,034	REF	REF
Currently married	8928	1.047 [0.994,1.103]	0.966 [0.860,1.086]
Risk population group			
GEN POP	4352	REF	REF
MSM	4780	1.866^***^[1.693,2.056]	2.143^***^[1.752,2.620]
FSW	17479	2.085^***^[1.921,2.263]	2.498^***^[2.121,2.941]
SDC	3658	2.729^***^[2.469,3.016]	3.097^***^ [2.607,3.679]
AGYW	2693	0.835^**^[0.736,0.947]	1.002 [0.801,1.253]
Prior PrEP Use			
No	31,106	REF	REF
Yes	1135	1.157^*^[1.020,1.312]	1.068[0.892,1.278]
STI diagnosis at visit 1			
No	32,189	REF	REF
Yes	206	1.341^*^[1.012,1.776]	1.262[0.865,1.841]

95% confidence intervals in brackets.

* *p* < 0.05,

** *p* < 0.01,

*** *p* < 0.001.

Adjusted for sub‐national geographic region and STI diagnosis at Visit 1.

Time observed in program = number of days between a client's first visit and the end of the observation period.

aOR, adjusted odds ratio; cOR, crude odds ratio; 95%CI, 95% confidence interval.

**Table 4b:** Crude and adjusted ordinal regression of factors associated with cycle number (Lesotho/TSEPO)

	No. of clients	cOR [95%CI]	aOR [95% CI]
Time observed in program	8510	1.003^***^[1.003,1.004]	1.003^***^[1.002,1.004]
Cycle length	8510	1.006^***^[1.004,1.006]	1.005^***^[1.004,1.006]
Age			
15—19	2254	REF	REF
20—24	2831	0.854[0.692,1.055]	1.097[0.864,1.392]
25—29	1491	0.990[0.776,1.262]	1.333 [0.928,1.914]
30—35	880	1.449^**^[1.116,1.882]	1.900^**^[1.281,2.817]
35 and over	1057	2.141^***^[1.708,2.683]	2.787^***^[1.908,4.072]
Sex			
Male	2546	REF	REF
Female	5967	0.829^*^[0.708,0.970]	1.192[0.912,1.556]
Marital status			
Not married	6393	REF	REF
Currently married	1217	1.097[0.892,1.351]	0.176[0.907,1.527]
Risk population group			
GEN POP	3791	REF	REF
MSM	399	2.037^***^[1.530,2.712]	3.342^***^[2.263,4.935]
FSW	365	1.307[0.927,1.843]	1.874^*^[1.137,3.088]
SDC	261	1.594^*^[1.100,2.314]	0.854[0.545,1.338]
AGYW	3752	0.842^*^[0.714,0.993]	1.511^*^[1.064,2.145]
STI diagnosis at visit 1			
No	5101	REF	REF
Yes	3407	0.325^***^[0.270,0.392]	0.423^***^[0.325,0.552]

95% confidence intervals in brackets.

* *p* < 0.05,

** *p* < 0.01,

*** *p* < 0.001.

Adjusted for sub‐national geographic region and STI diagnosis at Visit 1.

Time observed in program = number of days between a client's first visit and the end of the observation period.

Note: No clients had prior use of PrEP.

aOR, adjusted odds ratio; cOR, crude odds ratio; 95%CI, 95% confidence interval.

**Table 4c:** Crude and adjusted ordinal regression of factors associated with cycle number (Tanzania/Sauti)

	No. of clients	OR [95% CI]	B [95% CI]
Time observed in program	6059	1.004^***^[1.004,1.005]	1.003^***^[1.003,1.004]
Cycle length	6059	1.006^***^[1.005,1.007]	1.003^***^[1.002,1.005]
Age			
15—19	619	REF	REF
20—24	1817	1.046[0.850,1.288]	1.092[0.865,1.377]
25—29	1620	0.896[0.725,1.109]	0.965 [0.762,1.223]
30—35	957	0.911[0.722,1.150]	1.013[0.781,1.314]
35 and over	1046	0.924[0.735,1.162]	1.132[0.871,1.471]
Sex			
Male	434	REF	REF
Female	5626	22.055^***^[10.427, 46.652]	10.243^***^[4.755,22.065]
Marital status			
Not married	5878	REF	REF
Currently married	20	0.507[0.149, 1.725]	0.305[0.093,1.006]
Risk population group			
MSM	434	REF	REF
FSW	5624	22.047^***^[10.425, 46.623]	‐

95% confidence intervals in brackets.

* *p* < 0.05,

** *p* < 0.01,

*** *p* < 0.001.

Adjusted for sub‐national geographic region and STI diagnosis at Visit 1.

Time observed in program = number of days between a client's first visit and the end of the observation period.

Note: No clients had prior use of PrEP.

aOR, adjusted odds ratio; cOR, crude odds ratio; 95%CI, 95% confidence interval.

With each increase in cycle number, clients were 16% (14–18%) less likely in Kenya, 32% (15–46%) less likely in Lesotho and 47% (29–61%) less likely in Tanzania to stay off of PrEP an extra day (Table 5a, 5b, 5c). In other words, as cycle number increased, clients were more likely to restart PrEP sooner. Other factors associated with the likelihood of staying off PrEP for an extra month are outlined in Table 5a, 5b, 5c.

**Table 5a:** Crude and adjusted ordinal logistic regression of factors associated with the gaps (in days) between all cycles (Kenya/Jilinde)

	No. of clients	cOR [95% CI]	aOR [95% CI]
Cycle number	16,382	0.900^***^[0.882,0.919]	0.843^***^[0.824,0.863]
Time observed in program	16,382	1.001^***^[1.001, 1.001]	1.001^***^[1.001,1.001]
Cycle length	16,382	0.996^***^[0.996,0.997]	0.997^***^[0.996,0.997]
Age			
15—19	1,569	REF	REF
20—24	4683	1.044[0.939,1.161]	1.072 [0.959,1.198]
25–29	4136	0.934[0.839,1.040]	0.999 [0.890,1.122]
30‐35	2802	0.875^*^[0.780,0.981]	1007 [0.890,1.138]
35 and over	3,192	0.777^***^[0.697,0.867]	0.950 [0.842,1.072]
Sex			
Male	4008	REF	REF
Female	12,374	1.057[0.989,1.130]	1.059[0.953,1.177]
Marital status			
Not married	11,598	REF	REF
Currently married	4784	0.773^***^[0.728,0.820]	1.003[0.916,1.099]
Risk population group			
GEN POP	1312	REF	REF
MSM	2343	1.342^***^[1.184,1.520]	1.383^***^[1.178,1.624]
FSW	9523	1.201^**^[1.083,1.333]	1.470^***^[1.288,1.676]
SDC	2653	0.832^**^[0.741,0.933]	0.935[0.826,1.057]
AGYW	551	0.884[0.753,1.038]	0.818^*^[0.674,0.993]
Prior PrEP Use			
No	15,322	REF	REF
Yes	682	1.023[0.892,1.174]	0.940[0.822,1.076]
STI Diagnosis at Visit 1			
No	15,958	REF	REF
Yes	135	1.070[0.782,1.465]	1.137[0.842,1.534]

95% confidence intervals in brackets;

* *p* < 0.05,

** *p* < 0.01,

*** *p* < 0.001

Adjusted for sub‐national geographic region. Time observed in program = number of days between a client's first visit and the end of the observation period.

aOR, adjusted odds ratio; cOR, crude odds ratio; 95%CI, 95% confidence interval.

**Table 5b:** Crude and adjusted ordinal logistic regression of factors associated with the gaps (in days) between all cycles (Lesotho/TSEPO)

	No. of clients	cOR [95% CI]	aOR [95% CI]
Cycle number	942	0.820^*^[0.703,0.957]	0.682^**^[0.547,0.851]
Time observed in program	942	1.003^***^[1.002,1.004]	1.005^***^[1.004,1.006]
Cycle length	942	0.996^**^[0.993,0.998]	0.996^**^[0.994,0.999]
Age			
15–19	202	REF	REF
20–24	238	0.898[0.651,1.238]	0.677^*^[0.469,0.977]
25–29	152	0.932[0.641,1.355]	0.394^***^[0.239,0.648]
30–35	134	1.020[0.689,1.510]	0.507^*^[0.298,0.863]
35 and over	216	0.758[0.549,1.046]	0.321^***^[0.195,0.529]
Sex			
Male	313	REF	REF
Female	629	0.891[0.699,1.137]	0.868[0.598,1.258]
Marital status			
Not married	719	REF	REF
Currently married	130	1.108[0.808,1.520]	1.272[0.886,1.826]
Risk population group			
GEN POP	441	REF	REF
MSM	81	0.704[0.481,1.032]	0.883[0.570,1.368]
FSW	53	1.652^*^[1.019,2.677]	1.650[0.926,2.943]
SDC	40	1.818[0.956,3.459]	1.667[0.580,4.792]
AGYW	327	0.795[0.622,1.017]	0.741[0.470,1.170]
STI diagnosis at visit 1			
No	774	REF	REF
Yes	168	0.558^***^[0.424,0.734]	0.475^**^[0.298,0.756]

95% confidence intervals in brackets;

* *p* < 0.05,

** *p* < 0.01,

*** *p* < 0.001

Adjusted for sub‐national geographic region. Time observed in program = number of days between a client's first visit and the end of the observation period.

aOR, adjusted odds ratio; cOR, crude odds ratio; 95%CI, 95% confidence interval.

*Note*. Prior PrEP use omitted to collinearity.

**Table 5c:** Crude and adjusted ordinal logistic regression of factors associated with the gaps (in days) between all cycles (Tanzania/Sauti)

	No. of clients	cOR [95% CI]	aOR [95% CI]
Cycle number	1711	0.529^***^[0.412,0.680]	0.531^***^[0.393,0.718]
Time observed in program	1711	1.000[0.999,1.001]	1.000[0.999,1.000]
Cycle length	1711	0.996^**^[0.994,0.999]	0.991^***^[0.988,0.993]
Age			
15–19	183	REF	REF
20–24	547	0.831[0.571,1.211]	0.603^**^[0.422,0.861]
25–29	427	0.711[0.485,1.042]	0.505^***^[0.351,0.727]
30–35	263	0.889[0.599,1.320]	0.685[0.462,1.019]
35 and over	291	0.964[0.656,1.418]	0.607^*^[0.415,0.888]
Sex			
Male	7	REF	REF
Female	1704	0.323^***^[0.205,0.507]	0.828[0.412,1.664]
Marital status			
Not married	1708	REF	REF
Currently married	3	0.349[0.020,6.051]	1.582[0.066,37.738]
Risk population group			
MSM	7	REF	REF
FSW	1704	0.323^***^[0.205,0.507]	OMITTED

95% confidence intervals in brackets;

* *p* < 0.05,

** *p* < 0.01,

*** *p* < 0.001

Adjusted for sub‐national geographic region. Time observed in program = number of days between a client's first visit and the end of the observation period.

aOR, adjusted odds ratio; cOR, crude odds ratio; 95%CI, 95% confidence interval.

Note: Prior PrEP use and Visit 1 STI status omitted due to collinearity.

According to the Andersen–Gill model (Table 6a and 6c), for each increase in cycle number, the hazard of dropping‐off a cycle was 1.36 (1.33–1.38) in Kenya, 0.75 (0.68–0.82) in Lesotho and 1.12 (1.02–1.23) in Tanzania. Other factors associated with the hazard of dropping‐off a cycle earlier versus later varied by country. Among risk groups in Kenya, SDC (0.82 [0.79–0.84]), AGYW (0.88 [0.84–0.92]), MSM (0.88 [0.84–0.91]) for and FSW (0.96 [0.93–0.99]) were less likely to drop‐off compared to the general population (Table 6a). In Lesotho, the hazard of dropping‐off a cycle was 1.15 [1.03–1.29] for SDC (Table 6b). In Tanzania, the hazard of dropping‐off a cycle was 0.69 [0.63–0.75] among FSW compared to MSM, and 0.50 [0.41–0.61] among clients who had a positive STI diagnosis at their first visit compared to those who did not (Table 6c). Age was another factor that varied by country. In Kenya, compared to 15‐ to 19‐year olds, the hazard of dropping‐off a cycle was 0.97 [0.95–0.99] in 20‐ to 24‐year olds; 0.94 [0.91–0.96] in 25‐ to 29‐year olds; 0.92 [0.89–0.94] in 30‐ to 34‐year olds; and, 0.89 [0.87–0.92] in clients 35 years and older (Table 6a). Compared to 15‐ to 19‐year olds in Lesotho, clients 20–24 years were more likely to drop‐off a cycle (1.1 [1.05–1.15]) while clients 35 years and older were less likely (0.84 [0.77–0.92]) to drop‐off a cycle (Table 6b). Additionally, the hazard of dropping‐off a cycle in Lesotho for clients with a positive STI diagnosis at their first visit versus those without was 1.18 [1.10–1.25] and for married clients compared to unmarried clients was 0.78 [0.73–0.84] (Table 6b).

**Table 6a:** Factors associated with time to drop‐off of from a cycle (Andersen–Gill model)—Kenya/Jilinde

	Hazard ratio [95% CI]
Cycle number	1.36^***^[1.33,1.38]
Time observed through last cycle	0.99^**^[0.99,0.99]
Age categories	
15–19	REF
20–24	0.97^*^[0.95,0.99]
25–29	0.94^***^[0.91,0.96]
30–34	0.92^***^[0.89,0.94]
35 and over	0.89^***^[0.87,0.92]
Risk population group	
GEN POP	REF
MSM	0.88^***^[0.84,0.91]
FSW	0.96^**^[0.93,0.99]
SDC	0.82^***^[0.79,0.84]
AGYW	0.88^***^[0.84,0.92]
Sex	
Male	REF
Female	1.01[0.98,1.05]
Marital status	
Not married	REF
Currently married	1.02^*^[1.00,1.05]
Prior PrEP use	
No	REF
Yes	0.99[0.96,1.03]
STI Diagnosis at visit 1	
No	REF
Yes	0.95[0.86,1.05]
Year of first visit	
2017	REF
2018	0.23^***^[0.23,0.25]
2019	0.052^***^[0.048,0.056]

Observations = 31,735;

* *p* < 0.05,

** *p* < 0.01,

*** *p* < 0.001.

Adjusted for sub‐national geographic region. Time observed through last cycle = number of observation days a client contributed by the end of each cycle.

**Table 6b:** Factors associated with time to drop‐off from a cycle (Andersen–Gill model)—Lesotho/TSEPO

	Hazard ratio [95% CI]
Cycle number	0.745^***^[0.675,0.822]
Time observed through last cycle	1.001^**^[1.000,1.002]
Age categories	
15–19	REF
20–24	1.1^***^[1.051,1.152]
25–29	0.94[0.87,1.01]]
30–34	0.92[0.85,1.01]
35 and over	0.84^***^[0.77,0.92]
Risk population group	
GEN POP	REF
MSM	0.99[0.90,1.09]
FSW	0.97[0.86,1.09]
SDC	1.15^*^[1.03,1.29]
AGYW	0.95[0.89,1.03]
Sex	
Male	REF
Female	1.04[0.97,1.11]
Marital status	
Not married	REF
Currently married	0.78^***^[0.73,0.84]
STI Diagnosis at visit 1	
No	REF
Yes	1.18^***^[1.10,1.25]
Year of first visit	
2017	REF
2018	1.17[0.74,1.85]
2019	1.56[0.95,2.57]

Observations = 7571;

* *p* < 0.05,

** *p* < 0.01,

*** *p* < 0.001.

Adjusted for sub‐national geographic region. Time observed through last cycle = number of observation days a client contributed by the end of each cycle.

Note: Prior PrEP Use omitted due to collinearity.

**Table 6c:** Factors associated with time to drop‐off from a cycle (Andersen–Gill model)–Tanzania/Sauti

	Hazard ratio [95% CI]
Cycle number	1.12^*^[1.02,1.23]
Time observed through last cycle	0.98^***^[0.98,0.98]
Age categories	
15–19	REF
20–24	1.02[0.95,1.10]
25–29	1.04[0.97,1.12]
30–34	1.05[0.97,1.14]
35 and over	1.01[0.93,1.10]
Risk population group	
MSM	REF
FSW	0.69^***^[0.63,0.75]
Marital status	
Not married	REF
Currently married	1.10[0.91,1.32]
STI Diagnosis at visit 1	
No	REF
Yes	0.50^***^[0.41,0.61]
Year of first visit	
2018	REF
2019	0.01^***^[0.01,0.02]

Observations = 5897;

* *p* < 0.05,

** *p* < 0.01,

*** *p* < 0.001.

Adjusted for sub‐national geographic region. Time observed through last cycle = number of observation days a client contributed by the end of each cycle.

Note: Sex and Prior PrEP Use omitted due to collinearity.

### 3.2 First use cycle only

In Kenya, there was an increased probability of returning among older clients (**≥**20 years) and among each of the high‐risk groups compared to the general population (Table 7). After accounting for their increased probability of returning, the older age groups (**≥**25 years) and the high‐risk groups except AGYW were less likely to return for a refill compared to a restart, in Kenya (Table 7). In Lesotho, the probability of returning versus not after the first start was lower for 20‐ to 24‐year olds and higher for those 30 years and older compared to clients who were 15–19 years old. (Table 7). The probability of returning was higher among currently married clients, as well as among MSM and AGYW compared to the general population. Clients with a positive STI diagnosis at their first visit had a lower probability of returning (Table 7). The same factors were associated with probabilities of returning for a refill versus a restart, after accounting for the probability of having returned (Table 7). Details regarding the elapsed duration between the first PrEP stop and restart are outlined in Table 3.

**Table 7:** Probability to return for the first visit after initiation and type of first return (refill vs. restart) using the Heckman–Probit model for Kenya/Jilinde and Lesotho/TSEPO

	Selection model (return vs. no return)		Outcome Model (refill vs. restart)	
	Heckman–Probit estimates Kenya, Jilinde	Heckman–Probit estimates Lesotho, TSEPO	Heckman–Probit estimates Kenya, Jilinde	Heckman–Probit estimates Lesotho, TSEPO
Age				
15–19	REF	REF	REF	REF
20–24	0.056^*^[0.012,0.10]	−0.10^*^[−0.18, −0.019]	−0.0084[−0.064,0.047]	−0.11^*^[−0.20, −0.026]
25–29	0.19^***^[0.14,0.24]	0.11[−0.016,0.23]	−0.068^*^[‐0.13,‐0.0047]	0.12[−0.0073,0.24]
30–34	0.22^***^[0.17,0.28]	0.26^***^[0.13,0.40]	−0.12^***^[0.18, −0.049]	0.25^***^[0.11,0.39]
35 and over	0.31^***^[0.26,0.37]	0.39^***^[0.26,0.52]	−0.091^*^[−0.16, −0.020]	0.34^***^[0.21,0.47]
Sex				
Male	OMITTED	OMITTED	REF	REF
Female			−0.014[−0.080,0.051]	0.014[−0.024,0.052]
Currently married				
No	REF	REF	REF	REF
Yes	0.0049[−0.039,0.049]	0.24^***^[0.16,0.33]	−0.025[−0.078,0.028]	0.22^***^[0.13,0.30]
Risk population groups				
GEN POP	REF	REF	REF	REF
MSM	0.46^***^[0.40,0.52]	0.19^*^[0.040,0.35]	−0.44^***^[−0.53, −0.36]	0.18^*^[0.023,0.34]
FSW	0.47^***^[0.41,0.52]	0.019[−0.16,0.19]	−0.51^***^[−0.59,‐0.44]	−0.058[−0.24,0.13]
SDC	0.59^***^[0.53,0.65]	−0.12[‐0.31,0.064]	−0.23^***^[‐0.31, −0.15]	−0.08[−0.27,0.11]
AGYW	0.41^***^[0.33,0.49]	0.17^***^[0.078,0.27]	0.0077[‐0.11,0.12]	0.16^**^[0.060,0.26]
STI status at Visit 1				
No	REF	REF	REF	REF
Yes	0.14[−0.040,0.32]	−0.30^***^[−0.39, −0.22]	0.14[−0.070,0.36]	−0.22^***^[−0.30, −0.13]
Constant	−0.80^***^[−0.86,‐0.73]	−0.55^***^[−0.67, −0.43]		
Rho	−0.93[−0.99, −0.49]	1.00[0.51,1.00]	REF	REF
Observations (N)	32053	7606	0.031[−0.051,0.11]	−0.11^*^[−0.20, −0.026]

From the ordinal regression model (Table 8a), in Kenya, MSM were 55.4% [26.3–91.3%] and FSW were 54.8% [30.6–83.5%] more likely to stay off PrEP for an extra day compared to the general population (Table 8a). In Lesotho, compared to 15‐ to 19‐year olds, 20‐ to 24‐year olds, 25‐ to 29‐year olds, 30‐ to 35‐year olds, and clients 35 and older were 43.5% [16.7–61.7%], 66.2% [41.8–80.7%], 62.2% [27.8–80.2%] and 71.7% [51.0–86.7%] less likely to stay off PrEP for an extra day before returning for their first follow‐up, respectively (Table 8b). FSW were more than twice as likely (111.7% [54.0–325.0%]) to stay off PrEP for an extra day compared to the general population (Table 8b). A positive STI diagnosis at the first visit was associated with a 59.4% [31.3–74.8%] lower likelihood to stay off PrEP for an extra day compared to a negative STI diagnosis at the first visit (Table 8c). In Tanzania, 20‐ to 24‐year olds, 25‐ to 29‐year olds, 30‐ to 35‐year olds, and clients 35 and older were 48.7% [36.5–54.3%], 57.8% [38.8–70.9%], 44.0% [25.6–62.8%] and 46.9% [21.5–64.1%] less likely to stay off PrEP for an extra day before their first follow‐up, respectively, compared to clients who were 15–19 years old (Table 8c).

**Table 8a:** Crude and adjusted ordinal logistic regression of factors associated with the cycle gap duration in days between first stop and restart (Kenya/Jilinde)

	No. of clients	cOR [95% CI]	aOR [95% CI]
Time observed in program	9725	1.003^***^[1.003, 1.003]	1.001^***^[1.001,1.001]
Cycle length	9725	0.996^***^[0.996,0.997]	0.997^***^[0.996,0.997]
Age			
15‐19	1093	REF	REF
20‐24	2998	1.030[0.910,1.165]	1.063[0.933,1.211]
25‐29	2398	0.977[0.860,1.110]	1.022[0.888,1.175]
30‐35	1545	0.931[0.810,1.070]	1.067[0.917,1.240]
35 and over	1691	0.871^*^[0.762,0.995]	1.080[0.931,1.252]
Sex			
Male	2338	REF	REF
Female	7387	1.051[0.968,1.142]	1.095[0.949,1.263]
Marital Status			
Not married	7091	REF	REF
Currently Married	2634	0.734^***^[0.679,0.793]	0.962[0.853,1.085]
Risk Population Group			
GEN POP	1312	REF	REF
MSM	2343	1.355^***^[1.160,1.584]	1.554^***^[1.263,1.913]
FSW	9523	1.218^**^[1.067,1.390]	1.548^***^[1.306,1.835]
SDC	2653	0.766^**^[0.658,0.891]	0.912[0.777,1.070]
AGYW	551	0.784^*^[0.651,0.944]	0.793[0.627,1.005]
Prior PrEP Use			
No	9164	REF	REF
Yes	353	1.055[0.873,1.277]	0.949[0.785,1.147]
STI Diagnosis at Visit 1			
No	9478	REF	REF
Yes	72	0.973[0.609,1.556]	1.003[0.624,1.611]

95% confidence intervals in brackets;

* *p* < 0.05,

** *p* < 0.01,

*** *p* < 0.001.

Time observed in program = number of days between a client's first visit and the end of the observation period.

Abbreviations: aOR, adjusted odds ratio; cOR, crude odds ratio; 95%CI, 95% confidence interval.

**Table 8b:** Crude and adjusted ordinal logistic regression of factors associated with the cycle gap duration in days between first stop and restart (Lesotho/TSEPO)

	No. of clients	cOR [95% CI]	aOR [95% CI]
Time observed in program	718	1.003^***^[1.002,1.004]	1.005^***^[1.003,1.006]
Cycle length	718	0.996^**^[0.003,0.998]	0.996^**^[0.994,0.999]
Age			
15–19	176	REF	REF
20–24	190	0.814[0.587,1.128]	0.565^**^[0.383,0.833]
25–29	112	0.896[0.592,1.356]	0.338^***^[0.193,0.592]
30–35	92	0.854[0.529,1.381]	0.378^**^[0.198,0.722]
35 and over	148	0.723[0.507,1.030]	0.283^***^[0.163,0.490]
Sex			
Male	237	REF	REF
Female	481	0.834[0.633,1.100]	0.667[0.424,1.049]
Marital status			
Not married	549	REF	REF
Currently married	101	1.081[0.761,1.535]	1.421[0.967,2.089]
Risk population group			
GEN POP	310	REF	REF
MSM	63	0.688[0.445,1.065]	0.843[0.506,1.405]
FSW	41	1.683[0.966,2.932]	2.117^*^[1.054,4.250]
SDC	31	1.457[0.669,3.176]	1.281[0.419,3.914]
AGYW	273	0.785[0.595,1.036]	0. 771[0.443,1.340]
STI Diagnosis at visit 1			
No	589	REF	REF
Yes	129	0.504^***^[0.370,0.688]	0.416^**^[0.252,0.687]

95% confidence intervals in brackets;

* *p* < 0.05,

** *p* < 0.01,

*** *p* < 0.001.

Note: Prior PrEP Use omitted due to collinearity.

Time observed in program = number of days between a client's first visit and the end of the observation period.

Abbreviations: aOR, adjusted odds ratio; cOR, crude odds ratio; 95%CI, 95% confidence interval.

**Table 8c:** Crude and adjusted ordinal logistic regression of factors associated with the cycle gap duration in the days between first stop and restart (Tanzania/Sauti)

	No. of clients	cOR [95% CI]	aOR [95% CI]
Time observed in program	1492	1.016^***^[1.013,1.018]	1.000[0.999,1.001]
Cycle length	1492	0.994^***^[0.992,0.997]	0.990^***^[0.987,0.993]
Age			
15–19	157	REF	REF
20–24	477	0.714[0.493,1.033]	0.513^***^[0.357,0.735]
25–29	381	0.585^**^[0.399,0.859]	0.422^***^[0.291,0.612]
30–35	227	0.646^*^[0.432,0.967]	0.560^**^[0.372,0.844]
35 and over	250	0.891[0.605,1.312]	0.531^**^[0.359,0.785]
Sex			
Male	7	REF	REF
Female	1485	0.324^***^(0.188, 0.558)	0.920[0.446, 1.897]
Marital status			
Not married	1489	REF	REF
Currently married	3	0.214[0.036, 1.266]	1.614[0.066, 39.749]
Risk population group			
MSM	7	REF	REF
FSW	1485	0.324^***^[0.188, 0.558]	OMITTED

95% confidence intervals in brackets;

* *p* < 0.05,

** *p* < 0.01,

*** *p* < 0.001.

Note: Risk Population Group, Prior PrEP Use and STI Diagnosis at Visit 1 omitted due to collinearity.

Time observed in program = number of days between a client's first visit and the end of the observation period.

Abbreviations: aOR, adjusted odds ratio; cOR, crude odds ratio; 95%CI, 95% confidence interval.

## 4 DISCUSSION

This research illustrates that oral PrEP is most commonly used dynamically and with improved consistency as use experience is gained. This study is the first of its kind to analyse routine health services data collected universally across all PEPFAR‐supported PrEP services to yield insights into a broad diversity of PrEP use patterns and predictors. These data have already contributed to efforts to evolve global thinking on PrEP measurement and definitions of success [34, 35].

We found that early discontinuation and restarting PrEP at a later date was indeed common among the diverse client pool. According to the WHO criterion, only 12.4% of PrEP users in our analyses successfully continued to use PrEP. Despite almost 9 out of 10 clients stopping PrEP earlier than 90 days, over one‐third (35.9%) of these did return for at least one follow‐on prescription, suggesting incremental changes may be indicative of routinized use over time, as evidenced by shorter durations off PrEP between uses. Discontinuation durations varied widely, as did the phenomenon of stopping and restarting multiple times. Significant commonalities, however, included an observed decrease in the time off PrEP as cyclical use increased, suggestive of normalization/stabilization over time. Otherwise, we found few similarities among the PrEP use patterns in comparisons across country programs, though the diversity in patterns could be readily characterized and quantified in each locality. With existing data and appropriate analysis capacity, countries, implementing partners, sites and individual prescribers could identify clients’ use characteristics that may warrant enhanced adherence and persistence assistance to optimize effective HIV prophylaxis.

These findings contribute knowledge critical to better understanding service delivery to enhance client‐cantered care. Part of this is revising the expectation of continuous, contiguous days’ use of PrEP as required in HIV treatment, programs which have also begun to appreciate the cyclical nature of engagement in HIV care, and instead embracing flexibility for PrEP services [36, 37, 38]. There are now multiple studies definitively showing that PrEP offered at population levels, even with imperfect use and regardless of epidemic type, will have a significant impact on reducing new infections [15, 16, 39, 40, 41, 42, 43]. To maximize the potential of oral PrEP and achieve impact on new infections, implementation efforts will need to expand flexible, accessible services on population levels. These attributes are also important in allowing for adaptations during the COVID pandemic and other unexpected events. Furthermore, implications of our findings extend to future PrEP products beyond just oral PrEP, since behavioural determinants of use will remain, even if use decisions become less frequent.

Our findings are limited by our use of prescription data as a proxy for actual PrEP use. Furthermore, we have no data upon which to gauge the periodicity of risk. Research studies exploring actual daily use and risk are warranted to characterize and associate non‐continuous use and non‐continuous risk. Factors unrelated to risk influencing sporadic use, for example, limited access, side effects, stigma, forgetfulness, prevention method switching, non‐conscious biases, etc., could help further understand use/non‐use that may attenuate HIV protection. Such investigations are on‐going in the Jilinde program in Kenya. Finally, we only analysed the demographic and clinical predictor variables, including STI diagnosis, as they were noted by providers at baseline, though most of these could have changed over time.

## 5 CONCLUSIONS

Longitudinal cohort M&E approaches are necessary to identify a wide array of PrEP use patterns, characterize these patterns and predict factors associated with real‐world use experiences. Since flexible PrEP use may also be effective, users’ decisions around stopping PrEP may be well founded and reflect even greater feasibility of PrEP implementation, since flexible use may still be impactful, as opposed to failure to meet prevention objectives. In deference to current global indicators described above, client counselling largely continues to reinforce only long‐term use, without consideration of periodic stopping and restarting, phenomena that also require support in the form of counselling and tailored services. Donors should support implementers to mine existing data and invest in implementation science to better understand actual use and risk patterns, their overlap and how best to support clients starting, stopping, restarting and switching among the growing number of biomedical prevention options. The varying PrEP use narratives identified contribute to a comprehensive self‐care strategy and will have impacts on global HIV incidence reduction.

## COMPETING INTERESTS

The authors declare no conflict of interest.

## AUTHORS’ CONTRIBUTIONS

Study conception and design: J.B.R., P.S., J.M., B.W., J.R., R.E., D.M. Acquisition of data: J.B.R., P.S., J.M., B.W., D.M., N.M.N. Analysis and interpretation of data: J.B.R., P.S., J.M., B.W., D.M. Drafting of the manuscript: J.B.R., P.S., D.M. Critical revisions: J.B.R., P.S., D.W., T.C., J.M., B.W., A.M., N.M.N., A.C., R.P., J.R., R.E., K.C., D.M. Approval of final manuscript: J.B.R., P.S., D.W., T.C., J.M., B.W., A.M., N.M.N., A.C., R.P., J.R., R.E., K.C., D.M.

## Supporting information


**Table S1**: Glossary of variables **Figure S1**: Kaplan‐Meier curve depicting clients' time to drop‐off from a cycle in the Kenya Jilinde program for different Risk Population categories. (a) = Time observed in program for cycle 1. (b) Time observed in program for 2 or more cycles **Figure S2**: Kaplan‐Meier curve depicting clients' time to drop‐off from a cycle in the Lesotho TSEPO's program for different Risk Population categories. (a) = Time observed in program for cycle 1. (b) Time observed in program for 2 or more cycles
