# Supplementary material for: HIV PrEP is more than ART‐lite: Longitudinal study of real‐world PrEP services data identifies missing measures meaningful to HIV prevention programming
Source: J Int AIDS Soc. 2021 Oct 14;24(10):e25827. doi: 10.1002/jia2.25827 (PMC8516366; doi:10.1002/jia2.25827)
Supplement: Supplementary file 1 — Table S1: Glossary of variables Figure S1: Kaplan‐Meier curve depicting clients' time to drop‐off from a cycle in the Kenya Jilinde program for different Risk Population categories. (a) = Time observed in program for cycle 1. (b) Time observed in program for 2 or more cycles Figure S2: Kaplan‐Meier curve depicting clients' time to drop‐off from a cycle in the Lesotho TSEPO's program for different Risk Population categories. (a) = Time observed in program for cycle 1. (b) Time observed in program for 2 or more cycles [file JIA2-24-e25827-s001.docx]

**HIV PrEP is More than ART-lite: Longitudinal Study of Real-world PrEP Services Data Identifies Missing Measures Meaningful to HIV Prevention Programming**

Jason Bailey Reed^1*§^, Prakriti Shrestha^2*^, Daniel Were^3^, Tafadzwa Chakare^4^, Jane Mutegi^3^, Brian Wakhutu^3^, Abednego Musau^3^, Nyane Matebello Nonyana^4^, Alice Christensen^5^, Rupa Patel^6^, Jessica Rodrigues^7^, Robyn Eakle^8^, Kelly Curran^1^, Diwakar Mohan^9^

1 Jhpiego, Baltimore, USA

2 Department of Surgery, Johns Hopkins School of Medicine, Baltimore, USA

3 Jhpiego, Nairobi, Kenya

4 Jhpiego, Maseru, Lesotho

5 Jhpiego, Dar es Salaam, Tanzania

6 Washington University, St. Louis USA

7 AVAC, NY USA

8 USAID, Washington DC USA

9 Department of International Health, Johns Hopkins Bloomberg School of Public Health, Baltimore, USA

**Supplementary Table 1. Glossary of variables**

| **Term** | **Definition** |
| --- | --- |
| Cycle Gap | # days between stop date of prior cycle and start date of subsequent cycle [changed from months to days] |
| Cycle Length | # of days between first visit in a cycle and last apparent use at the end of each cycle |
| Cycle Number | # of cycles per client |
| Drop-off | A delay of 15 or more days in returning for a follow-on prescription (or no return at all) |
| Initiation | Client’s first PrEP prescription from the program |
| Refill | Client’s on-time return for additional PrEP supply, defined as a return within 14 days of the calculated date a client would no longer have PrEP tablets |
| Restart | Client’s return visit after drop-off |
| Time Observed in Program | # days between initiation date and final observation date (February 2019 for KJ and TS and September, 2019 for LT) |
|  |  |
| Time Observed through Last Cycle | # days between initiation date and date of apparent final use at end of each use cycle |

**Supplementary Figure 1. Kaplan-Meier curve depicting clients’ time to drop-off from a cycle in the Kenya Jilinde program for different Risk Population categories.** (a) = Time observed in program for cycle 1. (b) Time observed in program for 2 or more cycles.

a)

b)

 

**Supplementary Figure 2. Kaplan-Meier curve depicting clients’ time to drop-off from a cycle in the Lesotho TSEPO’s program for different Risk Population categories.** (a) = Time observed in program for cycle 1. (b) Time observed in program for 2 or more cycles.

**a)**

**b)**
